# A Rare Case of Fungal Necrotising Otitis Externa Centred on the Left Temporomandibular Joint

**DOI:** 10.1155/2020/8874754

**Published:** 2020-11-06

**Authors:** A. Khan, E. Omakobia, S. Hasnie, R. Barton, P. Gopalan, V. Oktseloglou, I. Smith

**Affiliations:** ^1^Department of ENT, Bradford Teaching Hospitals NHS Foundation Trust, Bradford, UK; ^2^Department of Microbiology, Bradford Teaching Hospitals NHS Foundation Trust, Bradford, UK; ^3^Department of Microbiology, Leeds Teaching Hospitals NHS Trust, Leeds, UK; ^4^Department of Radiology, Bradford Teaching Hospitals NHS Foundation Trust, Bradford, UK; ^5^Department of Oral and Maxillofacial Surgery, Bradford Teaching Hospitals NHS Foundation Trust, Bradford, UK

## Abstract

**Introduction:**

Necrotising otitis externa (NOE) is a rare life-threatening complication of simple otitis externa which can be difficult to diagnose and manage. It is very rarely centred on the temporomandibular joint (TMJ). Fungi cause NOE in approximately 5–20% of patients, and a high index of suspicion is required for diagnosis, particularly when there is no improvement with prolonged topical and intravenous antibiotic therapy.

**Objective:**

To report a novel case of fungal NOE centred on the left TMJ in an immunocompromised adult male with a focus on investigations and optimal management. *Case Report*. A 67-year-old male with comorbid chronic renal impairment presented to our otolaryngology department with prolonged left otalgia and otorrhoea. Subsequent cross-sectional imaging demonstrated left NOE centred on the TMJ. Poor resolution with prolonged courses of systemic and topical anti-pseudomonal antibiotics prompted maxillofacial surgical input for left TMJ exploration, washout, and biopsy from the joint capsule. The causative organism was identified as *Aspergillus flavus* on PCR analysis. The patient was successfully treated with oral posaconazole and repeated topical insertions of amphotericin B-soaked ribbon gauze to the left ear. *Discussion*. A combination of various imaging modalities including CT, MRI, Tc-99, and gallium-67 are utilised in clinical practice both to diagnose NOE and subsequently monitor disease progression or resolution. Immunocompromised patients with confirmed fungal NOE may require a combination of treatments including surgical debridement and prolonged antifungal therapy for a number of months, if not lifelong, treatment. Initiating empirical antifungal therapy may be justified in some patients. However, this should be judged on a case-by-case basis and guided by discussion with the local microbiology and infectious diseases departments. However, there is no national guideline or consensus regarding treatment of these patients, especially in cases of fungal NOE.

## 1. Introduction

Necrotising otitis externa (NOE) is a rare life-threatening complication of otitis externa, affecting the skull base, mastoid, and temporal bones. The disease is seldom centred on the temporomandibular joint. Mardinger et al. have shown an involvement of the TMJ in only 14% of cases with a high mortality rate of 50% [1]. There has been an exponential increase seen in cases of NOE; recent analysis of Hospital Episodes Statistics data for England showed a six-fold increase in the number of cases from 1999 (*n* = 67) to 2013 (*n* = 421), likely due to an ageing population in the UK [2]. Diabetes mellitus is an important predisposing factor in up to 94% of patients. However, other causes of immunocompromise such as AIDS, haematological malignancy, and end-stage renal disease (ESRD) have also been implicated [3]. *Pseudomonas aeruginosa* is isolated from the external auditory canal on aural swab in 50% to 90% of cases with NOE [4]. Less frequently, other bacterial organisms can be identified including *Staphylococcus aureus*, *Staphylococcus epidermidis*, *Klebsiella*, and *Proteus mirabilis* in 5% to 20% of cases [5]. Fungi cause NOE in 5% to 20% of these patients, with *Aspergillus* species most commonly identified in 50% of cases, specifically *A. fumigatus, A. niger*, *and A. flavus* [6].

There is currently no universal guidance in the literature for diagnosis, treatment, or follow-up. In 1987, Cohen and Friedman attempted to list diagnostic criteria for NOE using obligatory and occasional categories outlined as follows (Table 1) [7]. However, these criteria overemphasise the role of diabetes mellitus and side-line other forms of immunosuppression which are also important to consider. These criteria also fail to highlight which imaging modalities should be utilised. Clinical diagnosis initially is difficult because the symptoms of otalgia and otorrhea overlap with simple uncomplicated acute otitis externa. However, pain out of proportion to examination findings is a hallmark of NOE. Any degree of immunosuppression should alert a high degree of clinical suspicion towards a possible diagnosis of fungal NOE [8]. In addition to this, otoscopy revealing polyps and granulation tissue, particularly at the bone-cartilage junction of the external auditory canal makes a strong case for NOE [9]. Fungal NOE should be suspected when there is little to no improvement with both topical and intravenous antibiotic therapies. Inappropriate anti-pseudomonal treatment can often cause delays, misdiagnosis, and secondary fungal overgrowths [10]. Various case studies have reported a higher number of complications associated with fungal NOE such as cranial nerve palsies, pseudoaneurysms in the internal maxillary artery, and temporomandibular joint destruction [1, 11, 12]. With the recent advances in treatment modalities, the mortality rate has substantially reduced from 50% in the pre-1960s to 10–20% [13]. Here, we report an unusual case of fungal NOE centred on the left TMJ in a 67-year-old male with comorbid chronic renal impairment. Although rare, mortality from fungal NOE is high as elucidated above. Since no universal guideline or consensus exists regarding optimal investigations and treatment of this condition, we have presented our practice and also reviewed the literature to shed further light and guide clinicians managing this important condition.

## 2. Case Presentation

A 67-year-old comorbid patient was referred to our otolaryngology outpatient clinic with a 2-week history of severe left unilateral otalgia and right sided wax impaction. Clinical examination revealed two small left tympanic membrane perforations and a polyp in the floor of the left external auditory canal with granulation tissue. Both ears were cleared out with microsuction, and the left ear was treated with topical antibiotics (framycetin/gramicidin/dexamethasone: three drops three times a day for a week). The patient had a background of profound bilateral hearing loss, for which he wore bilateral hearing aids. His past medical history included hypertension, arthritis, bipolar disorder, and advanced nephropathy (chronic kidney disease stage 5) secondary to lithium use. There was no history of recent trauma. He was treated as an outpatient by junior members of the ENT team for several months with various topical antibiotics including Sofradex, ciprofloxacin, and clotrimazole as well as Otocomb ointment and regular aural microsuction. However, following senior clinical review, he was later admitted to hospital due to severe unrelenting otalgia necessitating further investigations due to concerns regarding skull base osteomyelitis. Examination of the left ear revealed significant debris and fungal spores within the ear canal with yellow mucopurulent discharge which was swabbed and sent for microscopy, culture analysis, and sensitivity analysis. Following microsuction, an anterosuperior perforation and a further large posterior perforation with attic granulation could be seen. Examination of the contralateral right ear revealed wax only which was cleared with microsuction to reveal an intact but thickened tympanic membrane. Full cranial nerve examination was normal.

## 3. Investigations and Treatment

**Figure 1:**
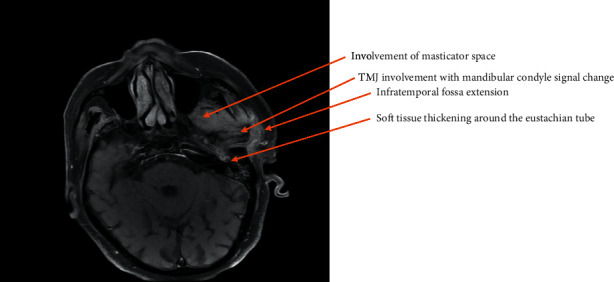
T1-weighted axial section MRI demonstrating left-sided necrotising otitis externa involving the left temporomandibular joint before treatment.

**Figure 2:**
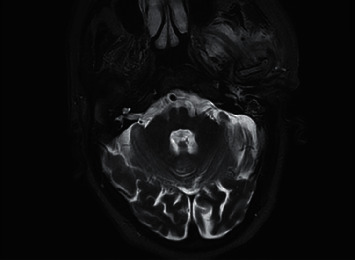
T2-weighted axial MRI prior to treatment being commenced for NOE again demonstrating inflammatory changes centred around the left temporomandibular joint.

**Figure 3:**
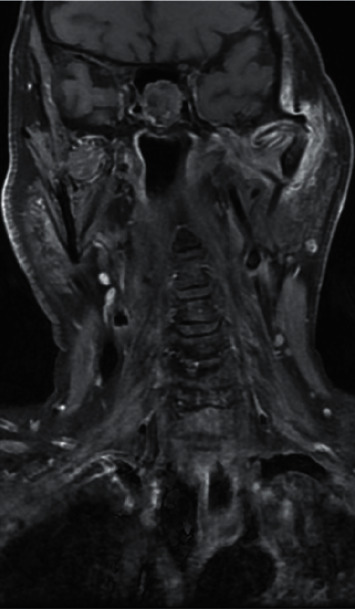
T1-weighted coronal section MRI showing abnormal soft tissue enhancement centred on the left TMJ.

Following admission to hospital, the patient was commenced on a three-month course of intravenous anti-pseudomonal antibiotics (ceftazidime, two grams three times daily), regular microsuction, and topical antifungal treatment (clotrimazole, two drops three times a day). *Aspergillus flavus* and *Candida albicans* were grown on an initial swab, but subsequent cultures were negative. Baseline observations showed the patient to be haemodynamically stable and apyrexial. Admission blood tests were unremarkable with a white cell count of 5.6 × 10^9^/L (normal range: 4–11 × 10^9^) and C-reactive protein of 11 mg/L (normal range: <5 mg/L). Due to unrelenting pain and progressive purulent otorrhea, he underwent further investigations. He was also subsequently switched to intravenous piperacillin/tazobactam (at a dose of 4.5 grams reduced to a twice daily dosing regimen due to renal impairment) and ciprofloxacin ear drops on advice from microbiology for five weeks. Computed tomography (CT) scans revealed inflammatory changes in the left masticator space with mastoid bone involvement suggestive of left necrotising otitis externa. Despite the above treatment, symptoms persisted, and magnetic resonance imaging (MRI) scanning revealed disease progression into the left TMJ and masticator space, prompting a maxillofacial surgical opinion (Figures 1–3). Microbiology also advised obtaining deep tissue samples to further guide treatment options. Following a joint washout and exploration of the left TMJ under general anaesthesia, a tissue biopsy from the joint capsule was taken, and all infected tissue was removed. This did not identify any causative microorganisms on microscopy and culture, but was, however, positive for DNA on panfungal PCR, and the sequence was identified as the *Aspergillus flavus* group. Microbiology advice was again sought with the recommendation to commence oral voriconazole. Following TMJ washout, the patient unfortunately developed left periorbital preseptal cellulitis which was treated with intravenous coamoxiclav. He also developed new-onset unilateral colour vision loss which coincided with the initiation of oral voriconazole. This was subsequently changed to oral posaconazole at a dose of 300 milligrams once daily along with repeated topical amphotericin-soaked ribbon wick insertions every 3 days into the left external auditory canal over a 3 week period. The patient was promptly referred to our infectious diseases team. Following their input, he was discharged home with oral posaconazole at the above dose and subsequently reviewed by both the ENT and infectious diseases teams as an outpatient.

### 3.1. Outcome and Follow-Up

**Figure 4:**
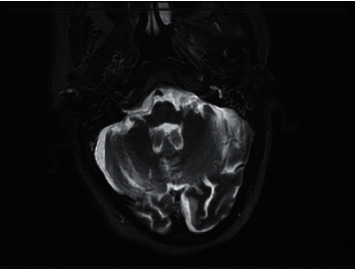
T2-weighted axial MRI following treatment: extensive signal changes in the left infratemporal fossa have resolved.

**Figure 5:**
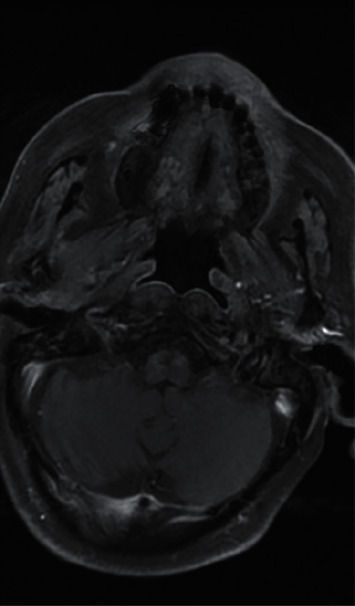
T1-weighted axial MRI slice showing abnormal soft tissue enhancement gradually resolved following six weeks of treatment.

The patient was reviewed in the ENT clinic six weeks following discharge, at which point he had completed just over 5 weeks of oral posaconazole. His otalgia had much improved, but unfortunately, he had undergone inpatient hospital admission under the medical team due to generalised lethargy, intermittent dysphagia, changes in voice, and episodes of choking. The cause of these symptoms was unclear following further assessment and investigations detailed as follows. Otoscopy on the right side was unremarkable apart from a small amount of wax. The left ear was cleared revealing inflammation and soft tissue thickening in the deep meatus. Flexible nasendoscopy revealed bilaterally mobile cords and a normal hypopharynx. Magnetic resonance imaging of the internal auditory meatus demonstrated good interval improvement, and the left otitis externa extending to the skull base had predominantly resolved (Figures 4 and 5). The scan highlighted a subtle focus of enhancement in the medulla which required further evaluation with an MRI brain scan with contrast. This further scan was normal. The patient was also referred to the community SALT (speech and language therapy) team. However, this was deferred due to the coronavirus pandemic. The patient was further reviewed by the infectious diseases team as an outpatient in February 2020 when he had completed just over 14 weeks of treatment, who decided to continue oral posaconazole (at a dose of 300 milligrams daily) due to its efficacy. He has since been followed up by the infectious diseases team in April 2020 completing 22 weeks of oral treatment, via a telephone consultation as he is currently shielding due to the COVID-19 pandemic. He remains well on oral posaconazole, having completing eight months of treatment thus far, and a follow-up MRI scan has been arranged as soon as the COVID-19 pandemic allows.

## 4. Discussion

Necrotising otitis externa is a rare but severe invasive infection of the external auditory canal which can spread rapidly to involve the surrounding tissue, adjacent neck spaces, and the skull base. A pathognomonic symptom differentiating NOE from simple otitis externa (swimmer's ear) can be pain out of proportion to clinical findings, especially overnight which can lead to disturbed sleep [14]. Susceptible patient groups are immunocompromised hosts, specifically elderly patients with diabetes mellitus and patients with HIV or haematological malignancies. It is most often caused by *Pseudomonas aeruginosa*. However, other causative agents include *Aspergillus, Staphylococcus aureus, Proteus mirabilis, Klebsiella oxytoca, Burkholderia cepacia*, and *Candida parapsilosis* [15]. It is often precipitated by iatrogenic trauma to the external auditory canal such as aural irrigation which could have been a factor in this case study, as the patient originally underwent microsuction to remove wax from both ear canals [16]. This may have caused local trauma, microangiopathy, and local hypoperfusion leading to an invasive fungal infection in an already immunocompromised host [17]. Other trauma can be self-inflicted such as the manipulation of the ear canal when using cotton buds and high-chloride environments such as swimming pool water [18].

Patients frequently present with exquisite otalgia that worsens at night, purulent otorrhea, difficulty with mastication, and hearing loss. It is thought that those who present with nasopharyngeal soft tissue involvement, cranial neuropathies, specifically facial nerve palsy, may have a poorer prognosis [19]. Intracranial complications are the most frequent cause of mortality in these cases [20]. Examination findings can vary but generally include oedema of the external ear canal, granulation tissue specifically at the site of Santorini's fissure (vertical fissures in the anterior part of the external auditory canal at the bone-cartilaginous junction), aural polyps, and foul-smelling purulent debris. Diagnosis requires microbiological sampling of ear debris or deep tissue sampling, CT and MRI scanning, and, in selected cases, technetium Tc-99 m and gallium scanning. Technetium and gallium scans can be useful for serial follow-up imaging to look for disease resolution or progression [21]. Immediate management of NOE requires hospital admission, treatment with systemic and topical antimicrobial therapy, regular microsuction, and, in certain cases, surgical debridement. Fungal NOE is an exceedingly rare opportunistic infection, which should be considered if the patient fails to respond to prolonged antibiotic therapy akin to this case.

A study of 42 patients with NOE over an 8-year period showed only 6 patients (14%) developed complications involving the TMJ [1]. Of these 6 patients, 4 were immunocompromised due to complications with diabetes but all six grew cultures for either *Pseudomonas aeruginosa, Staphylococcus epidermis, Aspergillus or Proteus mirabilis*. 3 eventually died of the disease, and the other 3 had uneventful healing, but the study concluded surgical debridement of the TMJ was necessary once the pathogenic organism was identified in case of further osteomyelitic bony destruction of the skull base. Another study, highlighting the importance of surgical intervention in treating polymicrobial infections of the temporal bone, identified four patients, three of which had invasive fungi as pathogens [22]. It concluded that immunocompromised patients may require a combination of treatments including surgical debridement and prolonged oral antifungal therapy for a number of months, if not lifelong treatment [22]. Aspergillus was implicated as the most common causative fungal organism in the literature, with treatment options being amphotericin B, voriconazole, fluconazole, posaconazole, and itraconazole [23]. The side effect profiles for voriconazole include visual disturbances, deranged electrolytes, and reduced renal function. In this case, the patient complained of unilateral colour vision loss which prompted a change in treatment to oral posaconazole, and a short-term topical treatment involving amphotericin B-soaked ribbon wick changes every 48 to 72 hours. There is no clear guidance on length of antifungal treatment or empirical use of antifungal therapy in intractable cases of bacterial NOE. There is limited evidence for the use of hyperbaric oxygen therapy; a recent study in Tunisia showed a faster rate of recovery for patients compared to antimicrobial therapy alone [24]. However, a Cochrane review in 2005 found inconclusive evidence to support the use of hyperbaric oxygen [25]. Another important aspect of management is control of underlying immunocompromised states, for example, strict glycaemic control for diabetics, low viral load, and high CD4 counts for patients with HIV and stable optimised renal function for those with chronic kidney disease. Regular radiological imaging is needed to assess treatment response once the patient has been discharged and is reviewed in an outpatient setting. The Cohen–Friedman criteria advocates technetium-99 (Tc-99) scintigraphy bone scanning as the gold standard, as it is more sensitive for NOE but lacks specificity for infective infiltration versus soft tissue inflammation [7]. Clinicians traditionally use computed tomography (CT) imaging alone for rapid initial assessment of the extension of disease and often use magnetic resonance imaging (MRI) later on to reveal the extent of soft tissue involvement, bone marrow infiltration, and intracranial involvement. A combination of various imaging modalities are utilised in clinical practice to diagnose and assess the severity of disease. CT, MRI, and Tc-99 imaging may have limited use when following up patients in an outpatient setting as changes on these scanning modalities can persist despite successful treatment [26]. Still many hospitals use CT and MRI scans at regular three-month intervals to assess treatment response [27]. Gallium-67 scans show positive uptake during active disease process as gallium binds with bacteria and lactoferrin from leucocytes making this sensitive and specific for detecting soft tissue and bone inflammation secondary to infection. However, it is expensive, and the restricted anatomic detail makes this imaging modality less effective in the initial assessment. It can, however, be more useful in monitoring treatment response [28].

This case study highlights the importance of considering fungal pathogens in case of NOE in patients who are unresponsive to antibiotic therapy. As in the reported case, deep tissue sampling is often required as negative cultures following prolonged antibiotic treatment can mask or propagate an invasive fungal infection which may progress into the skull base with potentially catastrophic consequences.

## 5. Conclusion

Fungal NOE remains poorly treated as there is limited guidance on antifungal choice and duration of treatment. It should always be considered and deep tissue sampling attempted, particularly in immunocompromised patients with intractable cases of NOE. Surgical debridement may be necessary in patients who have developed complications of their NOE such as destruction of the TMJ. Initiating empirical antifungal therapy may be justified in these patients. However, this should be judged on a case-by-case basis and guided by discussion with the local microbiology and infectious diseases departments.

## Figures and Tables

**Table 1:** Diagnostic criteria of NOE adapted from Cohen & Friedman.

Obligatory (major criteria)	Occasional (minor criteria)
Pain	*Pseudomonas* in culture
Exudate	Diabetes mellitus
Oedema	Elderly
Granulations	Cranial neuropathies
Micro abscesses (when operated)	Positive radiograph
Positive technetium-99 bone scan of failure of local treatment after more than 1 week	Debilitating conditions

All obligatory criteria must be met for diagnosis to be made.

## Data Availability

The data for this case were accessed through the electronic medical record. They are not available for readers to review as they contain confidential patient health information.

## References

[1] Mardinger O., Rosen D., Minkow B., Tulzinsky Z., Ophir D., Hirshberg A. (2003). Temporomandibular joint involvement in malignant external otitis. *Oral Surgery, Oral Medicine, Oral Pathology, Oral Radiology, and Endodontology*.

[2] Chawdhary G., Liow N., Democratis J., Whiteside O. (2015). Necrotising (malignant) otitis externa in the UK: a growing problem. Review of five cases and analysis of national hospital episode statistics trends. *The Journal of Laryngology & Otology*.

[3] Timon C. I., O’Dwyer T. (1989). Diagnosis, complications, and treatment of malignant otitis externa. *Irish Medical Journal*.

[4] Grandis J. R., Branstetter B. F., Yu V. L. (2004). The changing face of malignant (necrotising) external otitis: clinical, radiological, and anatomic correlations. *The Lancet Infectious Diseases*.

[5] Yang T.-H., Kuo S.-T., Young Y.-H. (2006). Necrotizing external otitis in a patient caused by *Klebsiella pneumoniae*. *European Archives of Oto-Rhino-Laryngology*.

[6] Cunningham M., Yu V. L., Turner J., Curtin H. (1988). Necrotizing otitis externa due to Aspergillus in an immunocompetent patient. *Archives of Otolaryngology—Head and Neck Surgery*.

[7] Curtin D., Friedman P. (1987). The diagnostic criteria of malignant external otitis. *The Journal of Laryngology & Otology*.

[8] Sylvester M. J., Sanghvi S., Patel V. M., Eloy J. A., Ying Y.-L. M. (2017). Malignant otitis externa hospitalizations: analysis of patient characteristics. *The Laryngoscope*.

[9] Glikson E., Sagiv D., Wolf M., Shapira Y. (2017). Necrotizing otitis externa: diagnosis, treatment, and outcome in a case series. *Diagnostic Microbiology and Infectious Disease*.

[10] Schrader N., Isaacson G. (2003). Fungal otitis externa-its association with fluoroquinolone eardrops. *Pediatrics*.

[11] Mani N., Sudhoff H., Rajagopal S., Moffat D., Axon P. R. (2007). Cranial nerve involvement in malignant external otitis: implications for clinical outcome. *The Laryngoscope*.

[12] Moffat H. A., Ibrahim W. H., Yousaf Z., Abubeker I. Y., Kartha A. (2019). Fungal malignant otitis externa involves a cascade of complications culminating in pseudoaneurysm of internal maxillary artery: a case report. *American Journal of Case Reports*.

[13] Bhandary S., Karki P., Sinha B. K. (2002). Malignant otitis externa: a review. *Pacific Health Dialogue*.

[14] Halsey C., Lumley H., Luckit J. (2011). Necrotising external otitis caused by Aspergillus wentii: a case report. *Mycoses*.

[15] Bovo R., Benatti A., Ciorba A., Libanore M., Borrelli M., Martini A. (2012). Pseudomonas and Aspergillus interaction in malignant external otitis: risk of treatment failure. *Acta Otorhinolaryngologica Italica: Organo Ufficiale Della Societa Italiana Di Otorinolaringologia E Chirurgia Cervico-Facciale*.

[16] Guevara N., Mahdyoun P., Pulcini C., Raffaelli C., Gahide I., Castillo L. (2013). Initial management of necrotizing external otitis: errors to avoid. *European Annals of Otorhinolaryngology, Head and Neck Diseases*.

[17] Raffaelli T., Dai C., Wang Z. (2013). The misdiagnosis of external auditory canal carcinoma. *European Archives of Oto-Rhino-Laryngology*.

[18] Rosenfeld R. M., Schwartz S. R., Cannon C. R. (2006). Clinical practice guideline: acute otitis externa. *Otolaryngology, Head and Neck Surgery*.

[19] Walton J., Coulson C. (2014). Fungal malignant otitis externa with facial nerve palsy: tissue biopsy AIDS diagnosis. *Case Reports in Otolaryngology*.

[20] Nawas M. T., Daruwalla V. J., Spirer D., Micco A. G., Nemeth A. J. (2013). Complicated necrotizing otitis externa. *American Journal of Otolaryngology*.

[21] Muñoz A., Martínez-Chamorro E. (1998). Necrotizing external otitis caused byAspergillus fumigatus: computed tomography and high resolution magnetic resonance imaging in an AIDS patient. *The Journal of Laryngology & Otology*.

[22] Marzo S. J., Leonetti J. P. (2003). Invasive fungal and bacterial infections of the temporal bone. *The Laryngoscope*.

[23] Denning D. W., Tucker R. M., Hansen L. H., Stevens D. A. (1989). Treatment of invasive aspergillosis with itraconazole. *The American Journal of Medicine*.

[24] Mardassi A., Turki S., Lahiani R., Mbarek H., Benzarti S., Gharsallh H. (2016). Is there a real benefit of hyperbaric oxygenotherapy in the treatment of necrotizing otitis externa?. *La Tunisie Medicale*.

[25] Phillips J. S., Jones S. E. (2005). Hyperbaric oxygen as an adjuvant treatment for malignant otitis externa. *Cochrane Database of Systematic Reviews*.

[26] Okpala N. C., Siraj Q. H., Nilssen E., Pringle M. (2005). Radiological and radionuclide investigation of malignant otitis externa. *The Journal of Laryngology & Otology*.

[27] Pringle A. M. J. L., Van Der Meer W. L., Bothof R. J. P., Van Tilburg M., Van Tongeren J., Postma A. A. (2018). Advanced imaging techniques in skull base osteomyelitis due to malignant otitis externa. *Current Radiology Reports*.

[28] Paramsothy M., Khanijow V., Ong T. O. (1997). Use of gallium-67 in the assessment of response to antibiotic therapy in malignant otitis externa—a case report. *Singapore Medical Journal*.

